# Potentiation of Nerve Growth Factor-Induced Neurite Outgrowth in PC12 Cells by Ifenprodil: The Role of Sigma-1 and IP_3_ Receptors

**DOI:** 10.1371/journal.pone.0037989

**Published:** 2012-05-24

**Authors:** Tamaki Ishima, Kenji Hashimoto

**Affiliations:** Division of Clinical Neuroscience, Chiba University Center for Forensic Mental Health, Chiba, Japan; Indiana University School of Medicine, United States of America

## Abstract

In addition to both the α1 adrenergic receptor and *N*-methyl-D-aspartate (NMDA) receptor antagonists, ifenprodil binds to the sigma receptor subtypes 1 and 2. In this study, we examined the effects of ifenprodil on nerve growth factor (NGF)-induced neurite outgrowth in PC12 cells. Ifenprodil significantly potentiated NGF-induced neurite outgrowth, in a concentration-dependent manner. In contrast, the α1 adrenergic receptor antagonist, prazosin and the NMDA receptor NR2B antagonist, Ro 25-6981 did not alter NGF-induced neurite outgrowth. Potentiation of NGF-induced neurite outgrowth mediated by ifenprodil was significantly antagonized by co-administration of the selective sigma-1 receptor antagonist, NE-100, but not the sigma-2 receptor antagonist, SM-21. Similarly, ifenprodil enhanced NGF-induced neurite outgrowth was again significantly reduced by the inositol 1,4,5-triphosphate (IP_3_) receptor antagonists, xestospongin C and 2-aminoethoxydiphenyl borate (2-APB) treatment. Furthermore, BAPTA-AM, a chelator of intracellular Ca^2+^, blocked the effects of ifenprodil on NGF-induced neurite outgrowth, indicating the role of intracellular Ca^2+^ in the neurite outgrowth. These findings suggest that activation at sigma-1 receptors and subsequent interaction with IP_3_ receptors may mediate the pharmacological effects of ifenprodil on neurite outgrowth.

## Introduction

Recent findings reveal that the sigma-1 receptor is a novel endoplasmic reticulum (ER) chaperone, which regulates a variety of cellular functions, such as inositol 1,4,5-triphosphate (IP_3_) receptor-mediated Ca^2+^ signaling, ion channel firing, protein kinase location/activation, cellular redox, neurotransmitter release, inflammation, cellular differentiation, neuronal survival and synaptogenesis [1]–[4]. Accumulating evidence suggests that the sigma-1 receptor plays an important role in neuronal plasticity, a process implicated in the pathophysiology of neuropsychiatric diseases, such as Alzheimer's disease, major depressive disorder, and schizophrenia [1]–[15].

PC12 cells, a cell line derived from a pheochromocytoma of rat adrenal medulla, have been widely used as a model system for nerve growth factor (NGF)-induced neuronal differentiation. It has been reported that sigma-1 receptor agonists, such as (+)-pentazocine, imipramine, fluvoxamine, donepezil, and dehydroepiandrosterone-sulfate (DHEA-S), potentiate NGF-induced neurite outgrowth in PC12 cells, and that the selective sigma-1 receptor antagonist NE-100 significantly attenuated the efficacy of these drugs, suggesting a role for sigma-1 receptors in neuronal plasticity [16]–[18]. However, the precise cellular mechanisms of the sigma-2 receptor are unclear, as this receptor has not yet been cloned.

Ifenprodil (Cerocral®) has been used as a cerebral vasodilator in a limited number of countries, including Japan and France. It acts as a prototypical antagonist of the *N*-methyl-D-aspartate (NMDA) receptor, NR2B subunit [19], [20]. As well as binding to the α1 adrenergic receptor and NMDA receptor antagonists, ifenprodil also binds to sigma-1 and sigma-2 receptors [21]–[23]. Despite the current knowledge, there are no reports describing the effects of ifenprodil on neuronal plasticity. In addition, it remains unclear whether ifenprodil acts as an agonist or antagonist on the sigma receptor subtypes.

In this study, we examined whether ifenprodil could potentiate NGF-induced neurite outgrowth in PC12 cells. We also examined the roles of sigma receptor subtypes 1 and 2, the α1 adrenergic receptor, and the NR2B subtype of the NMDA receptor in ifenprodil mediated, NGF-induced neurite outgrowth. Moreover, we looked at the role of IP_3_ receptors on NGF-induced neurite outgrowth by ifenprodil, since sigma-1 receptor associated neuronal plasticity utilizes IP_3_ receptors [1], [2], [17], [18].

## Results

### Effects of ifenprodil on NGF-induced neurite outgrowth

**Figure 1:**
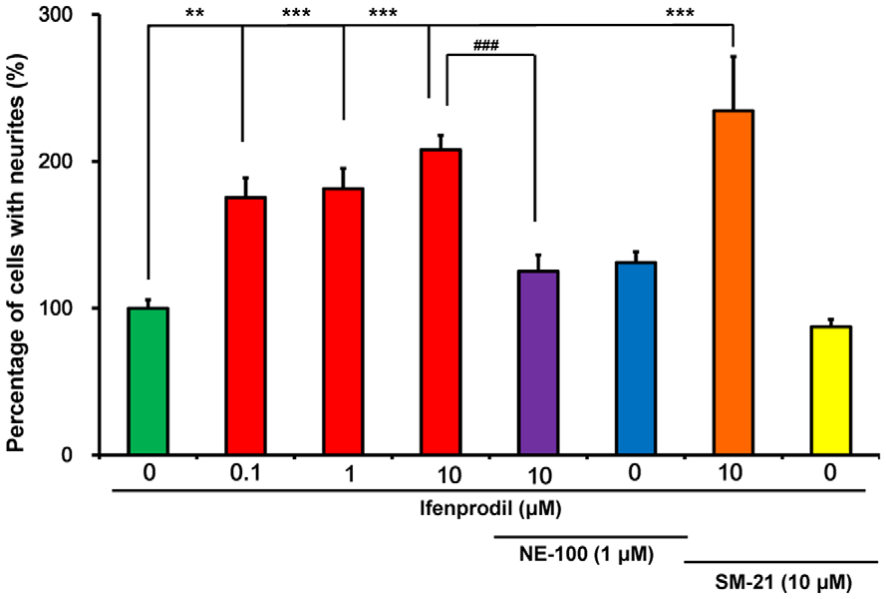
Effects of ifenprodil with or without sigma receptor antagonists on NGF-induced neurite outgrowth in PC12 cells. In the presence of NGF (2.5 ng/ml), ifenprodil (0.1, 1.0, or 10 µM) with or without NE-100 (1.0 µM; a sigma-1 receptor antagonist), or SM-21 (10 µM; a sigma-2 receptor antagonist) were incubated with PC12 cells. Four days after incubation with test drugs, morphometric analysis was performed. The data show the mean ± SEM (n = 6–18). **P<0.01, ***p<0.001 when compared to the control group. ^###^p<0.001 when compared to the ifenprodil (10 µM)-treated group.

**Figure 2:**
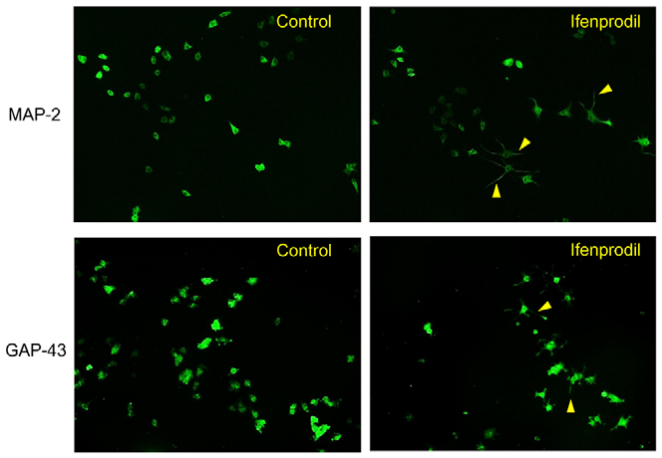
Representative photographs of MAP-2 and GAP-43 immunocytochemistry in PC12 cells. In the presence of NGF (2.5 ng/ml), vehicle control or ifenprodil (10 µM) were incubated with PC12 cells. Four days after incubation with test drugs, MAP-2 and GAP-43 immunocytochemistry was performed. Arrowhead shows the cells with neurite outgrowth.

Ifenprodil (0.1, 1.0, or 10 µM) increased the number of cells with neurite outgrowth in NGF (2.5 ng/ml) treated PC12 cells, in a concentration-dependent manner (**Figure 1**). MAP-2 and GAP-43 immunocytochemistry have been used as a useful indicator of neuronal differentiation in PC12 cells [17], [18], [24]. MAP-2 and GAP-43 immunocytochemistry revealed that the addition of ifenprodil (10 µM) increased the number of cells with neurite outgrowth in PC12 cells compared with controls (**Figure 2**).

### Role of sigma-1 receptor, but not α1 adrenergic receptor and the NMDA receptor, in the mechanisms of potentiation of NGF-induced neurite outgrowth by ifenprodil

In order to determine the role of sigma receptor subtypes, we examined the effects of NE-100 (a sigma-1 receptor antagonist) [25] and SM-21 (a sigma-2 receptor antagonist) [26] on the potentiation of NGF-induced neurite outgrowth by ifenprodil (10 µM). ANOVA analysis revealed that data from the eight groups differed significantly (F (7,64) = 17.46, p<0.001) (**Figure 1**). *Post hoc* Bonferroni/Dunn test results indicated that co-administration of NE-100 (1.0 µM) with ifenprodil (10 µM) significantly reduced the potentiation of NGF-induced neurite outgrowth (**Figure 1**). However, co-administration of SM-21 (10 µM) with ifenprodil (10 µM) did not affect the potentiation of NGF-induced neurite outgrowth (**Figure 1**). Administration of NE-100 (1.0 µM) or SM-21 (10 µM) alone did not alter NGF-induced neurite outgrowth in PC12 cells (**Figure 1**).

**Figure 3:**
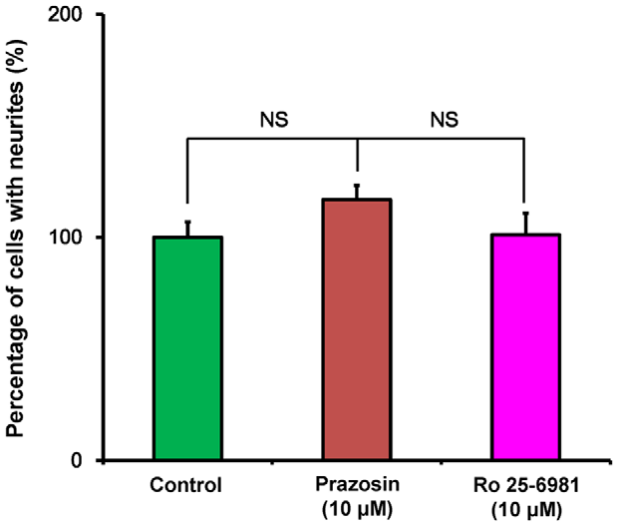
Effects of α1 adrenergic receptor antagonist and NR2B antagonist on NGF-induced neurite outgrowth in PC12 cells. In the presence of NGF (2.5 ng/ml), vehicle, prazosin (10 µM), or Ro 25-6981 (10 µM) were incubated with PC12 cells. Four days after incubation with test drugs, morphometric analysis was performed. The data show the mean ± SEM (n = 6). NS: Not significance.

To assess the role of the α1 adrenergic receptor and the NMDA receptor, NR2B subunit, we examined the effects of the selective α1 adrenergic receptor antagonist prazosin and the selective NR2B antagonist Ro 25-6981 [27] on the potentiation of NGF-induced neurite outgrowth by ifenprodil (10 µM). Neither the α1 adrenergic receptor antagonist, prazosin (10 µM) nor the NR2B antagonist Ro 25-6981 (10 µM) altered the number of cells with NGF induced neurite outgrowth (**Figure 3**), suggesting that these receptors do not play a role in the mechanisms of ifenprodil potentiation of neurite outgrowth.

### Role of IP_3_ receptor and intracellular Ca^2+^ in the mechanisms of potentiation of NGF-induced neurite outgrowth by ifenprodil

**Figure 4:**
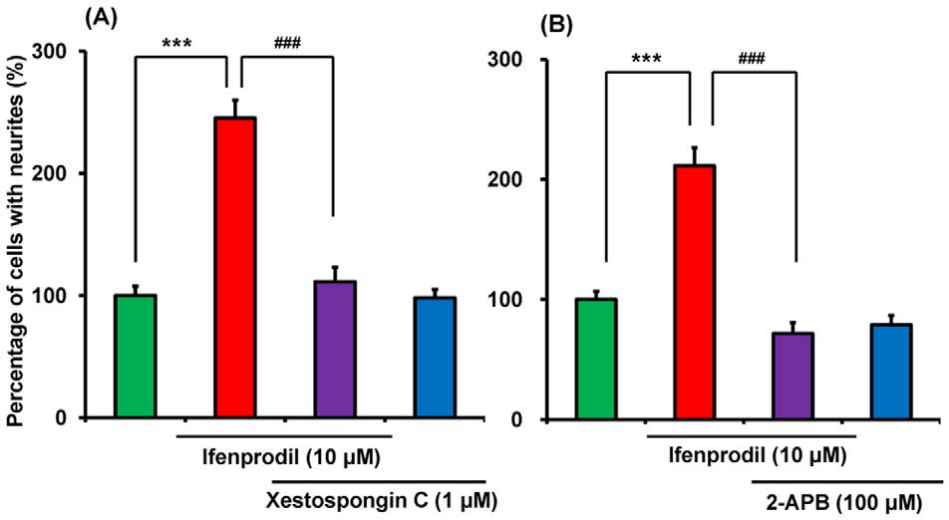
Effects of IP_3_ receptor antagonists on NGF-induced neurite outgrowth in PC12 cells. (A): In the presence of NGF (2.5 ng/ml), vehicle, ifenprodil (10 µM), ifenprodil (10 µM)+xestospongin C (1.0 µM), xestospongin C (1.0 µM) were incubated with PC12 cells. (B): In the presence of NGF (2.5 ng/ml), vehicle, ifenprodil (10 µM), ifenprodil (10 µM)+2-APB (100 µM), or 2-APB (100 µM) were incubated in PC12 cells. Four days after incubation with test drugs, morphometric analysis was performed. The data show the mean ± SEM (n = 6). ***p<0.001 when compared with the ifenprodil (10 µM)-treated group.

Next, we examined the effects of IP_3_ receptor antagonists, xestospongin C (a selective, reversible membrane-permeable inhibitor of IP_3_ receptors) [28] and 2-APB (a membrane-permeable IP_3_ receptor antagonist) [29], [30] on ifenprodil potentiation of neurite outgrowth. ANOVA analysis revealed significant differences among the four groups (F (3,20) = 44.02, p<0.001) (**Figure 4A**). Co-administration of xestospongin C (1.0 µM) significantly reduced neurite outgrowth by ifenprodil (10 µM) (**Figure 4A**). ANOVA analysis revealed that the data among the four groups differed significantly (F (3,20) = 40.52, p<0.001) (**Figure 4B**). Co-administration of 2-APB (100 µM) significantly reduced neurite outgrowth by ifenprodil (10 µM) (**Figure 4B**). Administration of xestospongin C (1.0 µM) or 2-APB (100 µM) alone did not alter NGF-induced neurite outgrowth in PC12 cells (**Figure 4A and 4B**).

**Figure 5:**
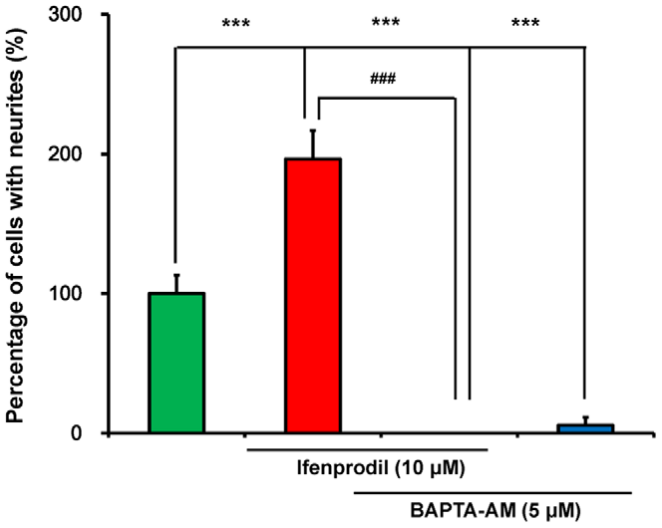
Effects of BAPTA-AM on potentiation of NGF-induced neurite outgrowth by ifenprodil. In the presence of NGF (2.5 ng/ml), vehicle, ifenprodil (10 µM), ifenprodil (10 µM)+BAPTA-AM (5.0 µM), or BAPTA-AM (5.0 µM) were incubated with PC12 cells. Four days after incubation with test drugs, morphometric analysis was performed. The data show the mean ± SEM (n = 6). ***p<0.001 when compared to the control group. ^###^p<0.001 when compared to the ifenprodil (10 µM)-treated group.

To assess the role of intracellular Ca^2+^ in the cells, we examined the effects of the BAPTA-AM, a chelator of intracellular Ca^2+^ [31], [32], on the potentiation of NGF-induced neurite outgrowth by ifenprodil (10 µM). ANOVA analysis revealed significant differences among the four groups (F (3,20) = 56.06, p<0.001) (**Figure 5**). Administration of BAPTA-AM (5.0 µM) significantly reduced neurite outgrowth by ifenprodil (10 µM) (**Figure 5**). In addition, BAPTA-AM (5.0 µM) alone significantly blocked NGF-induced neurite outgrowth. These findings suggest that the intracellular Ca^2+^ plays an important role in the mechanism of NGF-induced neurite outgrowth.

## Discussion

In this study, we found that ifenprodil potentiated NGF-induced neurite outgrowth in PC12 cells, and that the effects of ifenprodil were blocked by treatment with the selective sigma-1 receptor antagonist, NE-100 [25], but not the sigma-2 receptor antagonist, SM-21 [26]. Furthermore, the effects of ifenprodil were also blocked by treatment with two IP_3_ receptor antagonists. This is a first paper demonstrating a role for sigma-1 and IP_3_ receptors in ifenprodil mediated potentiation of NGF-induced neurite outgrowth. Recently, we reported that sigma-1 receptor agonists such as SA4503, fluvoxamine, DHEA-S and donepezil, could potentiate NGF-induced neurite outgrowth in PC12 cells, and that this potentiation could be antagonized by co-administration of NE-100 or xestospongin C [17], [18]. Taken together, it is likely that both sigma-1 and IP_3_ receptors are involved in the potentiation of NGF-induced neurite outgrowth by ifenprodil. In contrast, the sigma-2 receptor antagonist SM-21, failed to alter this enhanced outgrowth, suggesting a lack of involvement in this process.

It is likely that sigma-1 receptors interact with IP_3_ receptors on the ER, as well as regulate intracellular Ca^2+^ release. Recently, Hayashi and Su [1] identified sigma-1 receptors as novel, ligand-operated chaperones that specifically target the mitochondrion-associated ER membrane. When the sigma-1 receptor forms a complex with the ER chaperone, immunoglobulin heavy chain binding protein, BiP, activity is minimized. In contrast, when dissociated from BiP, the sigma-1 receptor exerts maximum chaperone activity. In addition, several synthetic agonists of the sigma-1 receptor promote its dissociation from BiP, thus stimulating the chaperone activity of this receptor [1]–[3]. In this study, we found that the cell-permeable Ca^2+^ chelator BAPTA-AM blocked the effects of ifenprodil on NGF-induced neurite outgrowth. In addition, BAPTA-AM alone blocked NGF-induced neurite outgrowth in PC12 cells, consistent with previous reports [31], [32]. These findings suggest that the intracellular Ca^2+^ plays an important role in the neurite outgrowth mediated by NGF. Therefore, it is likely that the therapeutic activity of sigma-1 receptor agonists, such as ifenprodil, could be mediated through modulation of intracellular Ca^2+^ signaling.

Recently, we reported that ifenprodil was effective in treating emotional incontinence in patients with vascular dementia (Kishimoto et al., submitted), and flashbacks in female post-traumatic stress disorder (PTSD) patients, with a history of childhood sexual abuse [33]. To our knowledge, this represents the first demonstration of a beneficial effect for ifenprodil in these groups of patients. However, the precise mechanisms underlying the therapeutic effects of ifenprodil are currently unclear. Given the role of sigma-1 receptors in modulating neurite outgrowth, it is likely that these receptors may at least play a partial a role in the beneficial effects of ifenprodil seen in these patients, although further detailed studies are needed. Accumulating evidence suggests a role for glutamatergic neurotransmission via the NMDA receptors, in the pathophysiology of PTSD [34], [35]. With its high affinity for both the NMDA and sigma-1 receptors, it is likely that ifenprodil acts on these receptors to alleviate emotional incontinence as well as flashbacks in these patients ([33], Kishimoto et al., submitted). Nonetheless, further large scale clinical studies will be needed to further support this initial finding.

In conclusion, this study suggests that ifenprodil can potentiate NGF-induced neurite outgrowth in PC12 cells, and that both the sigma-1 receptor and IP_3_ receptors play a role in the mechanisms of this potentiation. Therefore, it is likely that stimulation at sigma-1 receptors may be involved in the pharmacological action of ifenprodil in humans.

## Materials and Methods

### Drugs

The drugs listed here were used in these experiments: ifenprodil tartrate and prazosin (Sigma-Aldrich Co., Ltd., St. Louis, MO, USA), Ro 25-6981 maleate ((α*R*,β*S*)-α-(4-Hydroxyphenyl)-β-methyl-4-(phenylmethyl)-1-piperidinepropanol maleate) and SM-21 maleate ((±)-tropanyl 2-(4-chlorophenoxy)butanoate maleate) (Tocris Bioscience, Bristol, UK), xestospongin C (Wako Pure Chemical Industries, Tokyo, Japan), 2-aminoethoxydiphenyl borate (2-APB) (Calbiochem-Novabiochem Co., San Diego, CA, USA), NGF (Alomone Labs. Ltd., Jerusalem, Israel), BAPTA-AM, 1,2-bis(2-aminophenoxy)ethane-*N,N,N′,N′*-tetraacetic acid tetrakis) acetoxymethyl ester, (Dojindo Molecular Technologies, Inc., Kumamoto, Japan). The selective sigma-1 receptor antagonist NE-100 was synthesized in our laboratory. Other compounds were purchased from commercial sources.

### Cell culture and quantification of neurite outgrowth

PC12 cells (RIKEN Cell Bank, Tsukuba, Japan) were cultured at 37°C under 5% CO_2_ with Dulbecco's modified Eagle's medium (DMEM) supplemented with 5% heat-inactivated fetal bovine serum (FBS), 10% heat-inactivated horse serum, 1% penicillin and 1% streptomycin. The medium was changed two or three times a week. PC12 cells were plated onto 24-well tissue culture plates coated with poly-D-lysine/laminin. Cells were plated at relatively low density (0.25×10^4^ cells/cm^2^) in DMEM containing 0.5% FBS, 1% penicillin and 1% streptomycin. Medium containing a minimal level of serum (0.5% FBS) was used since serum contains steroid hormones which bind to sigma-1 receptors [16]–[18]. In a previous paper, we found that NGF at 2.5–40 ng/ml promoted neurite outgrowth in PC12 cells, in a concentration-dependent manner [17]. Therefore, NGF at a concentration of 2.5 ng/ml was used in this study. Twenty-four hours after plating, the medium was replaced with DMEM containing 0.5% FBS, 1% penicillin and 1% streptomycin with NGF (2.5 ng/ml), with or without ifenprodil (0.1, 1.0 or 10 µM), prazosin (α1 adrenergic receptor antagonist; 10 µM), Ro 25-6981 (NMDA receptor NR2B antagonist; 10 µM), NE-100 (sigma-1 receptor antagonist; 1.0 µM), SM-21 (sigma-2 receptor antagonist; 10 µM), xestospongin C (IP_3_ receptor antagonist; 1.0 µM), 2-APB (IP_3_ receptor antagonist; 100 µM) or BAPTA-AM (a chelator of intracellular Ca^2+^; 5.0 µM).

Four days after incubation with NGF (2.5 ng/ml) and test drugs, morphometric analysis was performed on digitized images of live cells, taken under phase contrast illumination, using an inverted microscope linked to a camera. Images of three fields per well were taken, with an average of 100 cells per field. The number of differentiated cells was determined by visually examining the field, and counting cells with at least one neurite equivalent to the length of a cell body diameter. This number was expressed as a percentage of the total number of cells in the field. Counting was performed in a blind manner as described previously [17], [18], [36], [37].

### MAP-2 and GAP-43 immunocytochemistry

Cells were fixed for 30 min at room temperature with 4% paraformaldehyde, then permeabilized with 0.2% Triton, and blocked with 5% normal goat serum, in 0.1 M phosphate-buffer saline containing 0.1% Tween-20 for 2 h. This was to reduce non-specific binding. Cells were incubated overnight at 4°C with anti-microtubule associated protein 2 (MAP-2) antibodies (1∶500 dilution in blocking solution; Chemicon International Inc., Temecula, CA, USA) or rabbit polyclonal antibody against growth-associated protein-43 (GAP-43) (1∶500 dilution in blocking solution; Abcam, Cambridge, MA, USA). Immunolabels were visualized using secondary antibodies conjugated to Alexa-488 (1∶1000; Invitrogen Corporation, Carlsbad, CA, USA).

### Statistical analysis

Data are expressed as the mean ± standard error of the mean (S.E.M.). Statistical analysis was performed by one-way analysis of variance (ANOVA) and *post hoc* Bonferroni/Dunn testing. Values of *p* less than 0.05 were considered statistically significant.
